# A clinical frailty scale obtained from MDT discussion performs poorly in assessing frailty in haemodialysis recipients

**DOI:** 10.1186/s12882-023-03126-0

**Published:** 2023-03-30

**Authors:** Benjamin M Anderson, Muhammad Qasim, Gonzalo Correa, Felicity Evison, Suzy Gallier, Charles J Ferro, Thomas A Jackson, Adnan Sharif

**Affiliations:** 1 Department of Nephrology and Transplantation, Queen Elizabeth Hospital, Edgbaston, B15 2WB Birmingham UK; 2 Institute of Inflammation and Ageing, University of Birmingham, Birmingham, UK; 3 Institute of Immunology and Immunotherapy, University of Birmingham, Birmingham, UK; 4 Department of Nephrology, Hospital del Salvador, Santiago, Chile; 5 Department of Health Informatics, Queen Elizabeth Hospital, Birmingham, UK; 6 PIONEER: HDR-UK hub in Acute Care, Edgbaston, Birmingham UK; 7 Institute of Cardiovascular Sciences, University of Birmingham, Birmingham, UK; 8 Department of Healthcare for Older People, Queen Elizabeth Hospital, Birmingham, UK

**Keywords:** Frailty, Haemodialysis, Mortality, Hospitalisation, Clinical Frailty Scale

## Abstract

**Background:**

The Clinical Frailty Scale (CFS) is a commonly utilised frailty screening tool that has been associated with hospitalisation and mortality in haemodialysis recipients, but is subject to heterogenous methodologies including subjective clinician opinion. The aims of this study were to (i) examine the accuracy of a subjective, multidisciplinary assessment of CFS at haemodialysis Quality Assurance (QA) meetings (CFS-MDT), compared with a standard CFS score via clinical interview, and (ii) ascertain the associations of these scores with hospitalisation and mortality.

**Methods:**

We performed a prospective cohort study of prevalent haemodialysis recipients linked to national datasets for outcomes including mortality and hospitalisation. Frailty was assessed using the CFS after structured clinical interview. The CFS-MDT was derived from consensus at haemodialysis QA meetings, involving dialysis nurses, dietitians, and nephrologists.

**Results:**

453 participants were followed-up for a median of 685 days (IQR 544–812), during which there were 96 (21.2%) deaths and 1136 hospitalisations shared between 327 (72.1%) participants. Frailty was identified in 246 (54.3%) participants via CFS, but only 120 (26.5%) via CFS-MDT. There was weak correlation (Spearman Rho 0.485, P < 0.001) on raw frailty scores and minimal agreement (Cohen’s κ = 0.274, P < 0.001) on categorisation of frail, vulnerable and robust between the CFS and CFS-MDT. Increasing frailty was associated with higher rates of hospitalisation for the CFS (IRR 1.26, 95% C.I. 1.17–1.36, P = 0.016) and CFS-MDT (IRR 1.10, 1.02–1.19, P = 0.02), but only the CFS-MDT was associated with nights spent in hospital (IRR 1.22, 95% C.I. 1.08–1.38, P = 0.001). Both scores were associated with mortality (CFS HR 1.31, 95% C.I. 1.09–1.57, P = 0.004; CFS-MDT HR 1.36, 95% C.I. 1.16–1.59, P < 0.001).

**Conclusions:**

Assessment of CFS is deeply affected by the underlying methodology, with the potential to profoundly affect decision-making. The CFS-MDT appears to be a weak alternative to conventional CFS. Standardisation of CFS use is of paramount importance in clinical and research practice in haemodialysis.

**Trial registration:**

Clinicaltrials.gov : NCT03071107 registered 06/03/2017.

**Supplementary Information:**

The online version contains supplementary material available at 10.1186/s12882-023-03126-0.

## Introduction

Frailty is the syndrome of accelerated ageing, characterised by multisystem dysregulation and increased vulnerability to stressors [1], and is associated with negative outcomes including mortality and hospitalization [2]. Frailty is common in haemodialysis recipients, but due to the lack of consensus on the optimal frailty instrument for kidney failure patients has been subject to heterogenous reporting. Our previous work has shown that estimates of frailty prevalence vary significantly between frailty tools within a haemodialysis population, ranging between 42 and 63% [3]. The simplest frailty tool is the Clinical Frailty Scale (CFS); [4] prevalence of frailty among prevalent haemodialysis patients in our cohort was 54% according to CFS, with weak agreement between frailty instruments on individual frailty status.

Other studies have also explored CFS use in haemodialysis patients. Frailty prevalence has been estimated at 26% using CFS in Canada, [5] derived from a subjective clinician judgement of frailty based upon available records, rather than as an adjunct to detailed clinical interview as originally validated [4] .

In haemodialysis patients, frailty is strongly associated with mortality [5–7] and increased hospitalisation rates [6–8]. Therefore, any consensus on the optimal frailty instrument in haemodialysis settings needs to consider ease of use and association with adverse outcomes for dialysis patients. While the CFS is simple to use, the level of agreement between the CFS obtained from subjective clinician judgment versus the more objective method based upon clinical interview is not known, despite both methods being used in clinical and research practice. Neither is it known whether these different methods of CFS acquisition affect the associations with negative outcomes for haemodialysis patients. It is important to compare the association of these two different methods of obtaining CFS with mortality and hospitalisation, to determine which methodology is best applied for use in haemodialysis recipients, and that was the aim of this analysis.

## Methods

### Study design

A detailed description of the FITNESS (**F**railty **I**ntervention **T**rial i**N E**nd-**S**tage patient**S** on haemodialysis) study has been reported [9]. Briefly, this first stage is a cross-sectional assessment and long-term follow up of study participants on maintenance haemodialysis with comprehensive frailty and bio-clinical phenotyping [10]. The study protocol was subject to favourable opinion by the South Birmingham Research Ethics Committee (Ref: 17/WM/0381) and institutional review board of University Hospitals Birmingham NHS Foundation Trust (RRK6082).

### Study setting

Patients were recruited from a single nephrology centre located in Birmingham, England. The service provides haemodialysis in a mixture of urban and rural settings, with a diverse range of ethnic and socioeconomic groups. Eligible patients were identified by interrogation of hospital electronic patient records (EPR) and discussion with clinicians at each dialysis unit. Eligible patients were approached, given written and verbal information about the study, and given sufficient opportunity to consider the information before giving consent for recruitment [3, 9]. The study was performed in accordance with the Declaration of Helsinki.

### Eligibility criteria

Inclusion criteria included adults aged ≥ 18, receiving regular haemodialysis for at least 3 months’ duration and ability to give informed consent. The only exclusion criterion was inpatient care within 4-weeks of recruitment unless for vascular access purposes, to avoid confounding of baseline data with frailty secondary to recent hospitalization [3, 9].

### Baseline assessment

Baseline assessments took place at individual dialysis units before and during their usual dialysis session. To negate potential effects of the long break from dialysis, we avoided the first haemodialysis session after the long weekend interval. In participants dialysing twice per week, the dialysis session after the shorter interdialytic interval was chosen for data collection.

Prior to connection to dialysis, participants underwent a timed walk over 4 m, Montreal Cognitive Assessment (MoCA) and grip strength dynamometer assessment (Takei Grip-D). Once dialysis started, a series of structured questionnaires were administered by the investigator team (henceforth “clinical interview”). These included demography, social history and frailty-specific questionnaires. Activities of daily living (ADL) and instrumental activities of daily living (IADL) [11, 12] were assessed by asking participants if they needed any help with the following: bathing, dressing, getting in or out of a chair, walking around the house, eating, grooming, using the toilet, getting up or down stairs, lifting 10lb (4.5 kg), meal preparation, shopping, taking medication, finances, housework, laundry, using the telephone, and transportation. These were recorded as binary “yes” or “no” responses. Investigators were permitted to seek clarification, and apply judgement in recording the responses in keeping with guidance from the CFS authors [13]. For example, if a participant never did the cooking in their household, this would not be recorded as dependency. EPR was interrogated for comorbidities, drug history, dialysis vintage/adequacy, previous transplantation and biochemical data. Determination of socio-economic deprivation was based upon the Index of Multiple Deprivation, a multiple deprivation model calculated at the local area level, with 1 representing the most deprived and 5 the least deprived area respectively [14].

### Frailty definitions

CFS was calculated using activity of daily living questionnaire results by the first author. CFS-MDT was ascertained as a result of MDT discussion at the participants’ monthly dialysis quality assurance (QA) meeting. Present at this QA meeting were the patients’ nephrologist, dialysis unit nurses, and dietitian; each had a copy of the CFS at the time of the meeting. CFS-MDT scores were arrived at by consensus from this MDT discussion; in the event of intractable MDT disagreement, the patients’ nephrologist had the casting vote on CFS-MDT frailty status. Frailty for both CFS and CFS-MDT was defined as a score of ≥ 5, vulnerability a score of 4 and robustness a score of ≤ 3. A score of 9 (end of life but not frail) was considered robust for analysis. These classifications were based in part upon our previous work comparing the CFS to other frailty scores [3], whilst aiming to maintain fidelity to the original descriptor terms for each level of CFS [13, 15].

### Outcomes

Mortality data were obtained by electronic record linkage of all FITNESS study recruits to Office of National Statistics (ONS), a UK-wide repository of death certificate data. This ensures robust coverage of mortality data capture and comprehensive description of causality. Electronic patient records, Hospital Episode Statistics (HES), and Office of National Statistics data were interrogated for hospitalisation. Admissions were defined as any hospital episode lasting ≥ 1 night. Primary diagnoses for each admission were recorded as these have been shown to be accurate for research purposes [19]. For ease of reporting and interpretation these were grouped into; (i) infection, (ii) dialysis access, (iii) dialysis indications/CKD 5 (e.g. fluid overload), (iv) cancer, and (v) major adverse cardiovascular events (MACE). Constituent HES codes are detailed in Supplementary Table 1. Transfers between hospitals were considered as one continuous admission, with the length of stay for such admissions considered to be the time from admission to the first hospital to discharge from the final hospital.

### Recruitment

A power calculation was originally performed based upon US data [16], assuming adjusted risk ratio of 2.24 for 1-year mortality and 1.56 for 1-year mortality and/or hospitalisation for frail versus non-frail patients receiving haemodialysis. A non-frail risk of 5% for 1-year mortality and a 40% risk of 1-year mortality/hospitalisation was assumed, powered to 0.8 and with a confidence interval of 0.95. A sample size of 602 was therefore considered to be robustly powered to demonstrate a difference in 1-year mortality or 150 patients to be powered for 1-year mortality/hospitalisation. However, in agreement with the sponsor, recruitment of 602 participants was not felt to be feasible in this single centre, and a revised target of 500 participants was set with follow up beyond 1-year.

### Statistics

Statistical analysis was performed using Stata (StataCorp 2019, Stata Statistical Software: Release 16. College Station, TX: StataCorp LLC) and R Studio (version 1.3.959). Categorical data was presented as numbers and percentages, with continuous variables reported as medians and interquartile ranges (IQRs).

Correlations were analysed using Spearman’s test. Agreement between frailty scores were analysed using Cohen’s Kappa. Strength of agreement was rated as > 0.90 almost perfect agreement, 0.80–0.90 strong, 0.60–0.79 moderate, 0.40–0.59 weak, 0.21–0.39 minimal and ≤0.20 no agreement [17].

Survival analyses were performed for both mortality and a composite of mortality/first hospitalisation. Survival analyses were performed by generation of Kaplan-Meier curve estimates and time-to-event outcomes were analysed with the Cox’s Proportional Hazards Model. The proportional hazard assumption was satisfied by examination of plots of the log-negative-log of the within-group survivorship functions versus log time as well as comparing Kaplan-Meier (observed) with Cox (expected) survival curves with our study variables, alongside selected covariables for adjusted analyses (reported as Hazard Ratios [HR] with 95% Confidence Intervals [CI]). Survival time was accrued from the day of baseline data collection and was censored for end of follow-up. Incidence rate ratios were obtained for count data by Negative Binomial Regression, offset by length of follow-up. Negative binomial distribution was confirmed by interrogation of means and variances and visual interpretation of expected versus observed distribution plots. All regressions were performed treating frailty both as continuous and ordinal (Robust, Vulnerable, or Frail) variables; these were performed unadjusted and adjusted for an *a priori* list of covariables, considered based on a known or suspected relationship with dialysis-related admission (age, sex, ethnicity, body mass index, index of multiple deprivation, Charlson comorbidity index [CKD omitted], number of hospitalisation episodes, polypharmacy, smoking status, serum albumin, use of walking aids, dialysis vintage and kidney transplant wait-listing in addition to frailty status).

Missing IMD Quintile data were handled via a dummy variable. All other missing data was assumed missing at random and handled via listwise deletion as all other covariables had < 1% data missing. A p value < 0.05 was considered significant in the statistical analysis.

## Results

**Fig. 1:**
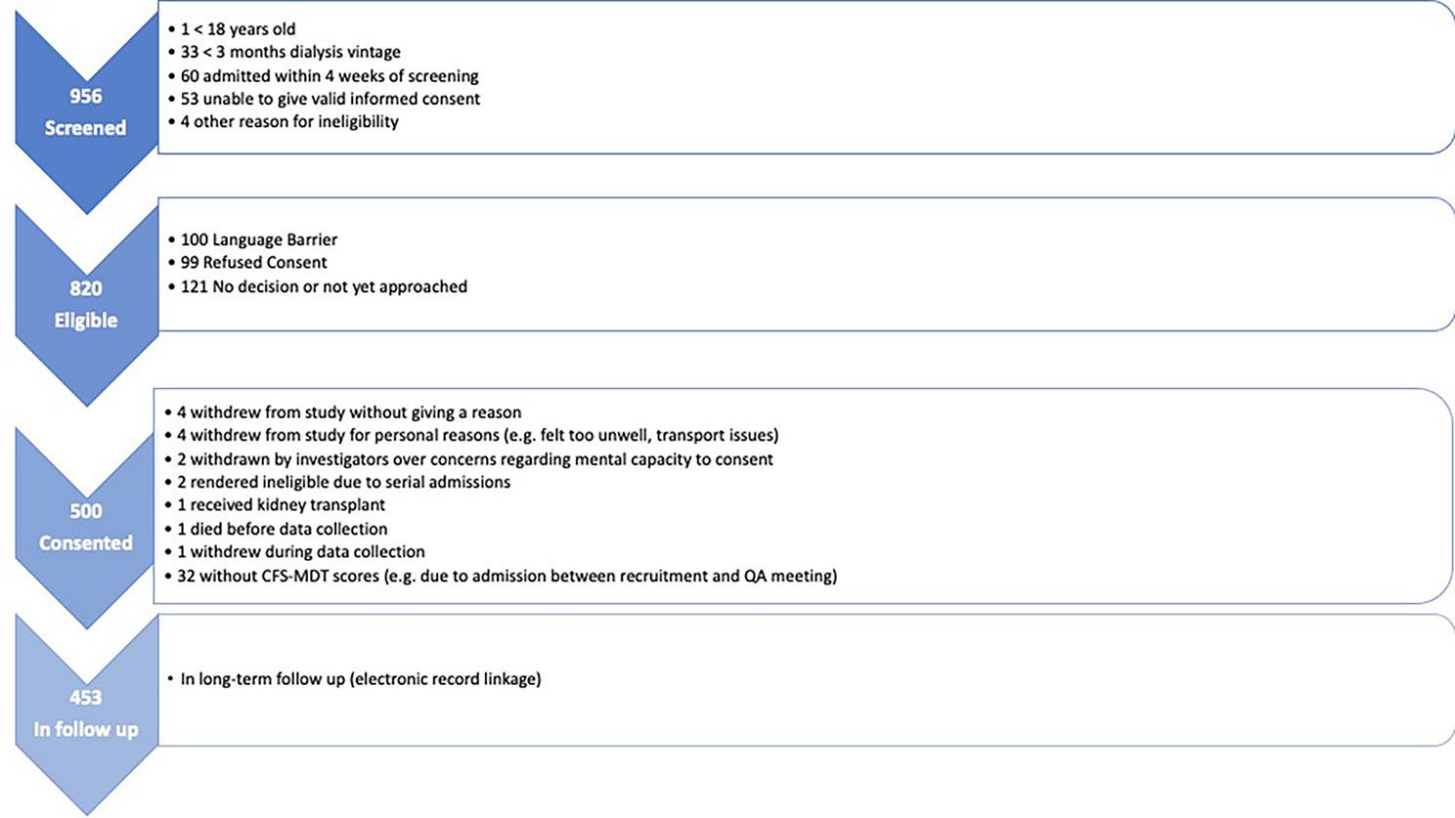
PRIMSA flow diagram of participant recruitment

A PRISMA diagram of recruitment is shown in Fig. 1. In total, 500 participants gave informed consent to participate in the cohort study between 12th January 2018 and 18th April 2019. After initial approach 15 participants withdrew from the study either prior to, or during data collection. A further 32 participants did not get discussed at the relevant dialysis QA (e.g., due to hospital admission after recruitment) and hence did not receive a CFS-MDT score. This left 453 participants with data available for this analysis. Table 1 shows baseline demographics and co-morbidities of study participants.

**Table 1:** Baseline characteristics of the FITNESS cohort, including those considered frail by CFS and CFS-MDT

Parameter	Total cohort (n = 453)	Participants adjudged as frail by:	
		CFS	CFS-MDT
Frail participants (% of total cohort)	-	246 (54.3%)	120 (26.5%)
Median Age*	63 (54–74)	65 (55–75)	70.5 (59-78.5)
*Ethnicity*			
White	263 (58.1%)	133 (54.1%)	70 (58.3%)
South Asian	107 (23.6%)	68 (27.6%)	31 (25.8%)
Black	72 (15.9%)	40 (16.3%)	19 (15.8%)
Other ethnicity	11 (2.4%)	5 (2.0%)	0 (0%)
Male	268 (59.2%)	131 (53.3)	61 (50.8%)
Median BMI*	26.9 (23.2–32.3)	27.9 (23.3–33.7)	27.4 (23.6–33.9)
*IMD Quintile*			
1	199 (43.9%)	111 (45.1%)	52 (43.3%)
2	79 (17.4%)	39 (15.9%)	19 (15.8%)
3	79 (17.4%)	45 (18.3%)	20 (16.7%)
4	36 (7.95%)	17 (6.9%)	11 (9.2%)
5	31 (6.8%)	17 (6.9%)	9 (7.5%)
Unknown	29 (6.4%)	17 (6.9%)	9 (7.5%)
Median Albumin*	39 (35–41)	38 (35–41)	37 (33–40)
Median MoCA Score*	22 (18–25)	20 (16–23)	19 (15–23)
*Cause of kidney failure*			
Diabetes	107 (23.6%)	74 (30.1%)	45 (37.5%)
Ischaemic	36 (8.0%)	22 (8.9%)	11 (9.2%)
Hypertensive	36 (8.0%)	17 (6.9%)	5 (4.2%)
IgA	35 (7.7%)	15 (6.1%)	6 (5.0%)
PKD	28 (6.2%)	11 (4.5%)	4 (3.3%)
FSGS	22 (4.9%)	10 (4.1%)	5 (4.2%)
Reflux	15 (3.3%)	10 (4.1%)	5 (4.2%)
Obstructive	16 (3.5%)	6 (2.44%)	5 (4.2%)
AAV	15 (3.3%)	4 (1.6%)	3 (2.5%)
Insterstitial Nephritis	10 (2.2%)	4 (1.6%)	1 (0.8%)
Myeloma	9 (2.0%)	2 (0.8%)	2 (1.7%)
Other	38 (8.4%)	23 (9.4%)	11 (9.2%)
Unknown	63 (13.9%)	35 (14.2%)	13 (10.8%)
*Medical co-morbidities*			
MI	87 (19.2%)	57 (23.2%)	30 (25.0%)
Heart failure	46 (10.2%)	32 (13.0%)	13 (10.8%)
Stroke/TIA	53 (11.7%)	36 (14.6%)	25 (20.7%)
PVD	41 (9.1%)	28 (11.4%)	20 (16.7%)
Cancer	53 (11.%)	26 (10.6%)	14 (11.7%)
*Smoking history*			
Current	63 (13.9%)	29 (11.8%)	13 (10.8%)
Previous	124 (27.4%)	64 (26.0%)	32 (26.7%)
Never	266 (58.7%)	153 (62.2%)	75 (62.5%)
Median Charlson score* **	4 (3–6)	5 (4–6)	6 (4.5-7)
*Dialysis details*			
Median dialysis vintage (months)	36.2 (17.5–75.6)	40.6 (19.9–79.0)	42.0 (22.0-87.7)
Line access	99 (21.9%)	60 (24.4%)	35 (29.2%)
Median Kt/V	1.60 (1.41–1.85)	1.62 (1.41–1.89)	1.60 (1.38–1.88)
*Transplant list status*			
Active	54 (11.9%)	22 (8.9%)	1 (0.8%)
Suspended	13 (2.9%)	6 (2.4%)	0 (0%)
Not listed	386 (85.2%)	218 (88.6%)	119 (99.2%)
Employment status			
Employed	62 (13.7%)	8 (3.3%)	2 (1.7%)
Unemployed	136 (30.0%)	84 (34.2%)	27 (22.5%)
Retired	255 (56.3%)	154 (62.6%)	91 (75.8%)
Job role***			
Unskilled Manual	172 (39.5%)	106 (45.9%)	49 (43.0%)
Skilled Manual	99 (22.7%)	50 (21.7%)	27 (23.7%)
Clerical	48 (11.0%)	20 (8.7%)	10 (8.8%)
Managerial	41 (9.4%)	19 (8.2%)	9 (7.9%)
Professional	76 (17.4%)	36 (15.6%)	19 (16.7)
Education level			
High School	322 (71.1%)	186 (75.6%)	98 (81.7)
College/Sixth Form	84 (18.5%)	39 (15.9%)	15 (12.5%)
University	47 (10.4%)	21 (8.5%)	7 (5.8%)
*Residence*			
Own home	431 (95.6%)	230 (93.9%)	108 (90.0%)
Warden-controlled flat	12 (2.7%)	9 (3.7%)	5 (4.2%)
Residential home	5 (1.1%)	3 (1.2%)	4 (3.3%)
Nursing home	3 (0.7%)	3 (1.2%)	3 (2.5%)
With professional carers****	32 (7.37)	32 (12.7%)	21 (18.8%)

All numbers shown n (%) except * which indicates median (IQR). Percentages shown within CFS and CFS-MDT columns indicate percentage of those considered frail by relevant instrument unless otherwise stated. ** = CKD excluded. *** = Current job role or previous if retired/unemployed. **** = if not in nursing or residential care

Participants were followed-up through EPR linkage for a median of 685 days (IQR 544–812) and all participants had a minimum potential follow-up of 365 days, barring mortality.

### Frailty prevalence, correlation and agreement

Using the CFS, 127 (28.0%) participants were classified as robust, 80 (17.7%) vulnerable, and 246 (54.3%) frail. By contrast, using the CFS-MDT 250 (55.2%) were classified as robust, 83 (18.3%) were vulnerable, and 120 (26.5% were frail).

**Fig. 2:**
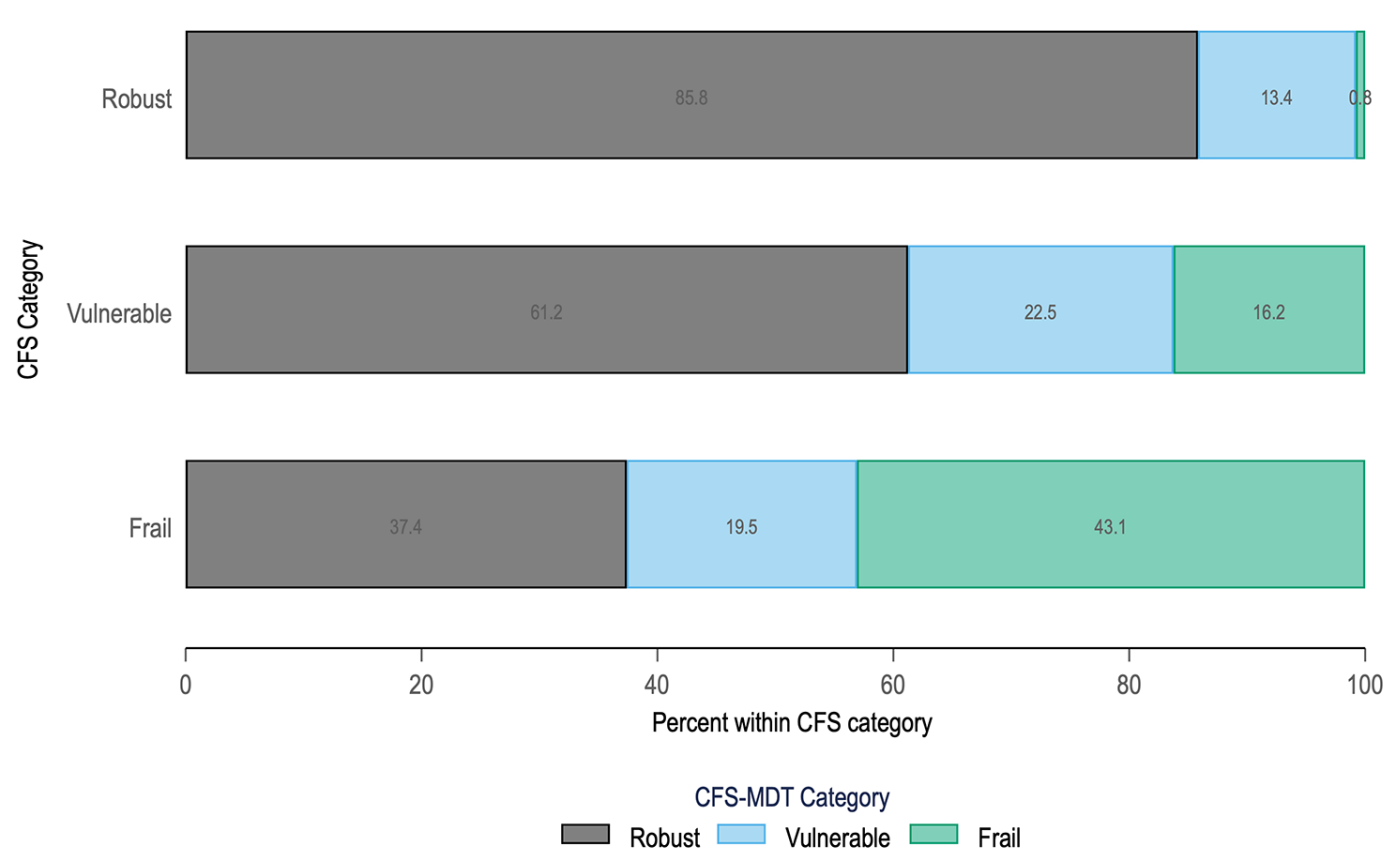
Comparison of frailty status between CFS and CFS-MDT

The CFS and CFS-MDT were weakly correlated (Spearman Rho 0.485, P < 0.001), and agreement was minimal upon splitting into ordinal categories of robust, vulnerable, and frail (Cohen’s κ = 0.274, P < 0.001). Figure 2 demonstrates substantial discrepancy in frailty status between the CFS and CFS-MDT. For example, 37.4% of participants adjudged frail by CFS were considered robust by the CFS-MDT. There was also substantial variation in both correlation and agreement between CFS and CFS-MDT depending upon dialysis unit, as shown in Table 2.

**Table 2:** Agreement between CFS and CFS-MDT divided by dialysis units

HD Unit	*κ*	P	${\rm{\rho }}$	P
1	**0.510**	**< 0.001**	**0.741**	**< 0.001**
2	0.053	0.134	0.204	0.174
3	**0.158**	**0.018**	**0.457**	**< 0.001**
4	**0.448**	**< 0.001**	**0.660**	**< 0.001**
5	**0.218**	**0.019**	**0.373**	**0.011**
6	0.076	0.153	**0.491**	**0.013**
7	**0.339**	**< 0.001**	**0.634**	**< 0.001**
8	**0.322**	**< 0.001**	**0.561**	**< 0.001**
9	**0.268**	**< 0.001**	**0.484**	**< 0001**
10	**0.470**	**< 0.001**	**0.512**	**0.013**

Figures shown derived from Cohen’s κ for agreement and Spearman’s ρ for correlation. Dialysis units anonymized

### Mortality

There were 96 deaths (21.2% of total) during follow-up. Infection was the primary cause of death for 20 (20.8%) participants, ischaemic heart disease 10 (10.4%), cerebrovascular disease 3 (3.1%), cancer 10 (10.4%), and CKD or haemodialysis complications in 3 (3.1%). Of the 20 infection-related deaths, 8 (8.3% of total deaths) were due to COVID-19. Cause of death data were not available for 20 (20.8%) participants. The most common causes of death are summarised in Supplementary Table 1.

**Fig. 3:**
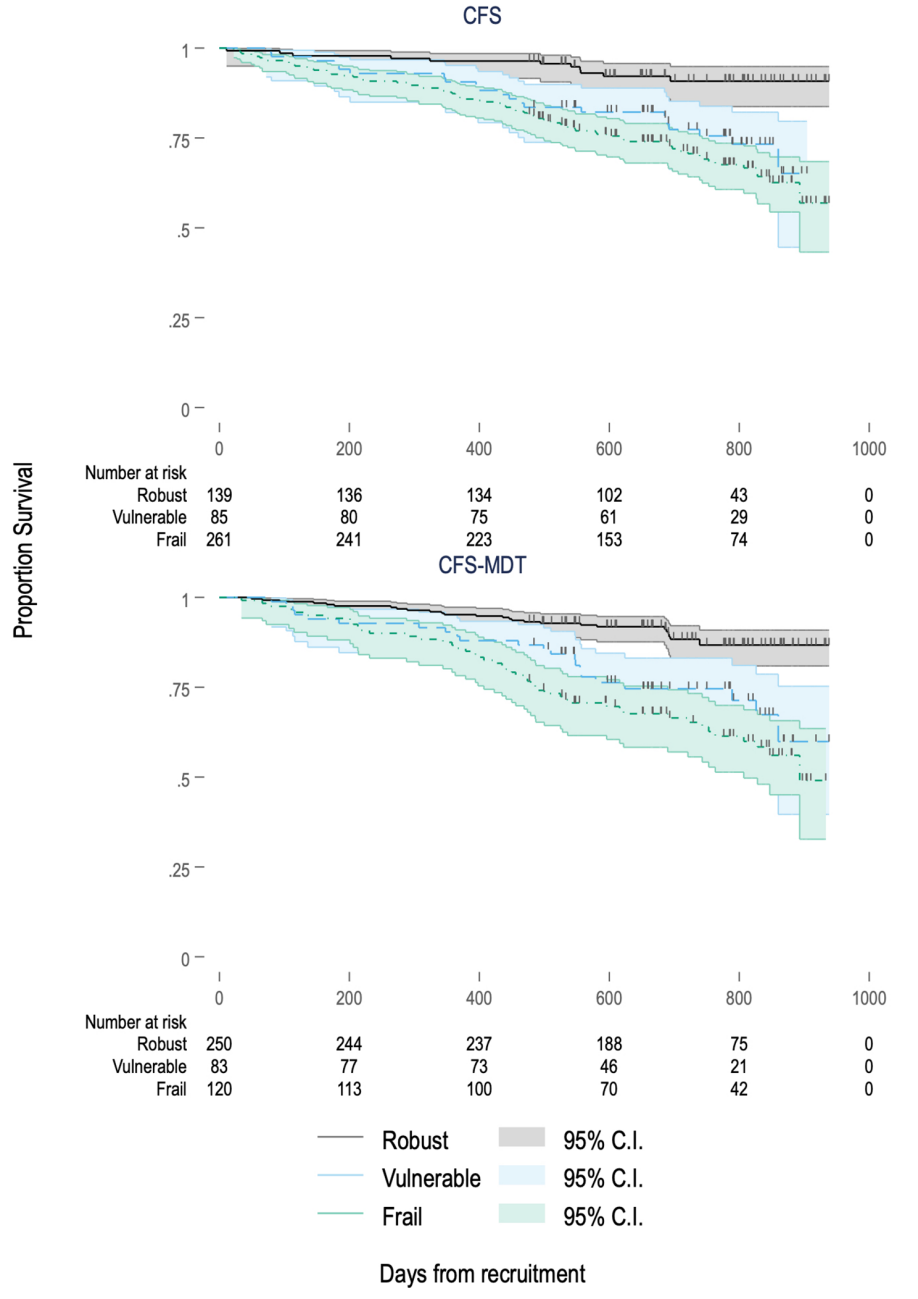
Kaplan-Meier curves of survival by frailty category for CFS and CFS-MDT

Figure 3 shows Kaplan-Meier curves for survival by frailty category for both frailty tools. Table 3 shows that increasing frailty via both CFS and CFS-MDT was associated with increasing hazard for mortality after adjustment for *a priori* covariables. When split into robust, vulnerable, or frail categories, both frailty and vulnerability were associated with greater hazard for mortality when compared with robustness on multivariable analysis, as shown in Table 4. Fully adjusted model results are detailed in Supplementary Tables 3–6.

**Table 3:** Hazard Ratios for mortality and mortality/hospitalisation by frailty tool when treated as a continuous variable

	CFS		CFS-MDT	
	HR	P	HR	P
	(95% C.I.)		(95% C.I.)	
*Mortality*				
Univariable	1.50	< 0.001	1.54	< 0.001
	(1.29–1.76)		(1.36–1.73)	
Multivariable	1.39	0.002	1.36	< 0.001
	(1.13–1.70)		(1.16–1.59)	
*Mortality/Hospitalisation*				
Univariable	1.19	< 0.001	1.18	< 0.001
	(1.10–1.29)		(1.10–1.27)	
Multivariable	1.14	0.01	1.12	0.019
	(1.03–1.26)		(1.02–1.23)	

Hazard Ratios and 95% confidence intervals shown. All significant at P < 0.05 level

**Table 4:** Hazard Ratios for mortality and mortality/hospitalisation for frailty when considered as a categorical variable

		CFS		CFS-MDT	
		HR	P	HR	P
		95% C.I.		95% C.I.	
Mortality					
Univariable	*Vulnerable*	**3.68**	**0.002**	**2.71**	**0.001**
		**1.60–8.47**		**1.54–4.76**	
	*Frail*	**5.03**	**< 0.001**	**3.93**	**< 0.001**
		**2.42–10.45**		**2.44–6.31**	
Multivariable	*Vulnerable*	**2.76**	**0.028**	**2.03**	**0.024**
		**1.12–6.83**		**1.10–3.77**	
	*Frail*	**3.60**	**0.003**	**2.30**	**0.004**
		**1.56–8.34**		**1.30–4.07**	
Mortality/Hospitalisation					
Univariable	*Vulnerable*	1.28	0.151	1.22	0.188
		0.91–1.80		0.91–1.63	
	*Frail*	**1.70**	**< 0.001**	**1.61**	**< 0.001**
		**1.30–2.21**		**1.26–2.06**	
Multivariable	*Vulnerable*	1.22	0.281	1.08	0.634
		0.85–1.75		0.79–1.48	
	Frail	**1.56**	**0.008**	1.35	0.058
		**1.13–2.16**		0.99–1.85	

Hazard Ratios and 95% confidence intervals shown. Bold text indicates significance at P < 0.05 level

### Mortality/hospitalisation

There were 1136 hospital admissions during follow-up, shared between 327 (72.1%) participants; the median number of admissions was 2 (IQR 0–4). Of these, 634 admissions occurred within the first year, split between 263 (58.1%) participants, with a median of 1 admission within 1-year of recruitment (IQR 0–2). Infection was listed as primary diagnosis in 254 (22.6%) admissions, 239 (21.3%) admissions were due to dialysis access, 139 (12.4%) for dialysis-specific indications, there were 41 (3.65%) MACE during admissions.

**Fig. 4:**
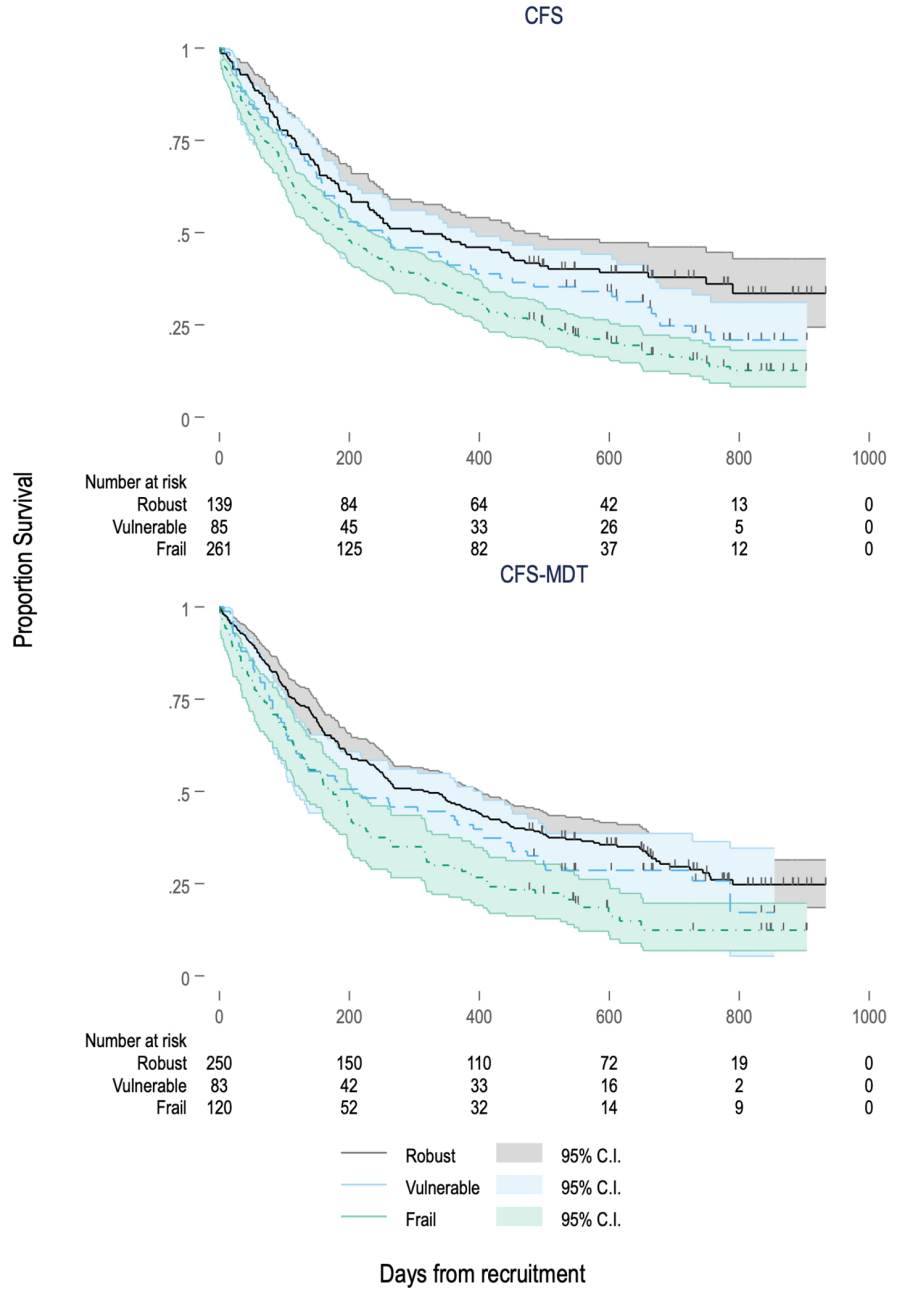
Kaplan-Meier curves of admission-free survival by frailty category for CFS and CFS-MDT

Figure 4 shows Kaplan-Meier curves for admission-free survival by frailty category for both frailty tools. Increasing frailty was associated with increased hazard for mortality/hospitalisation by both CFS and CFS-MDT on both unadjusted and adjusted analysis, as shown in Table 3. However, Table 4 shows that when split into categories, only frailty was associated with increased hazard of mortality/hospitalisation when compared with robustness for both CFS and CFS-MDT, and this lost significance for the CFS-MDT on adjusted analysis. Fully adjusted Cox regression models for mortality/hospitalisation are shown in Supplementary Tables S7-10.

### Rates of admission

**Fig. 5:**
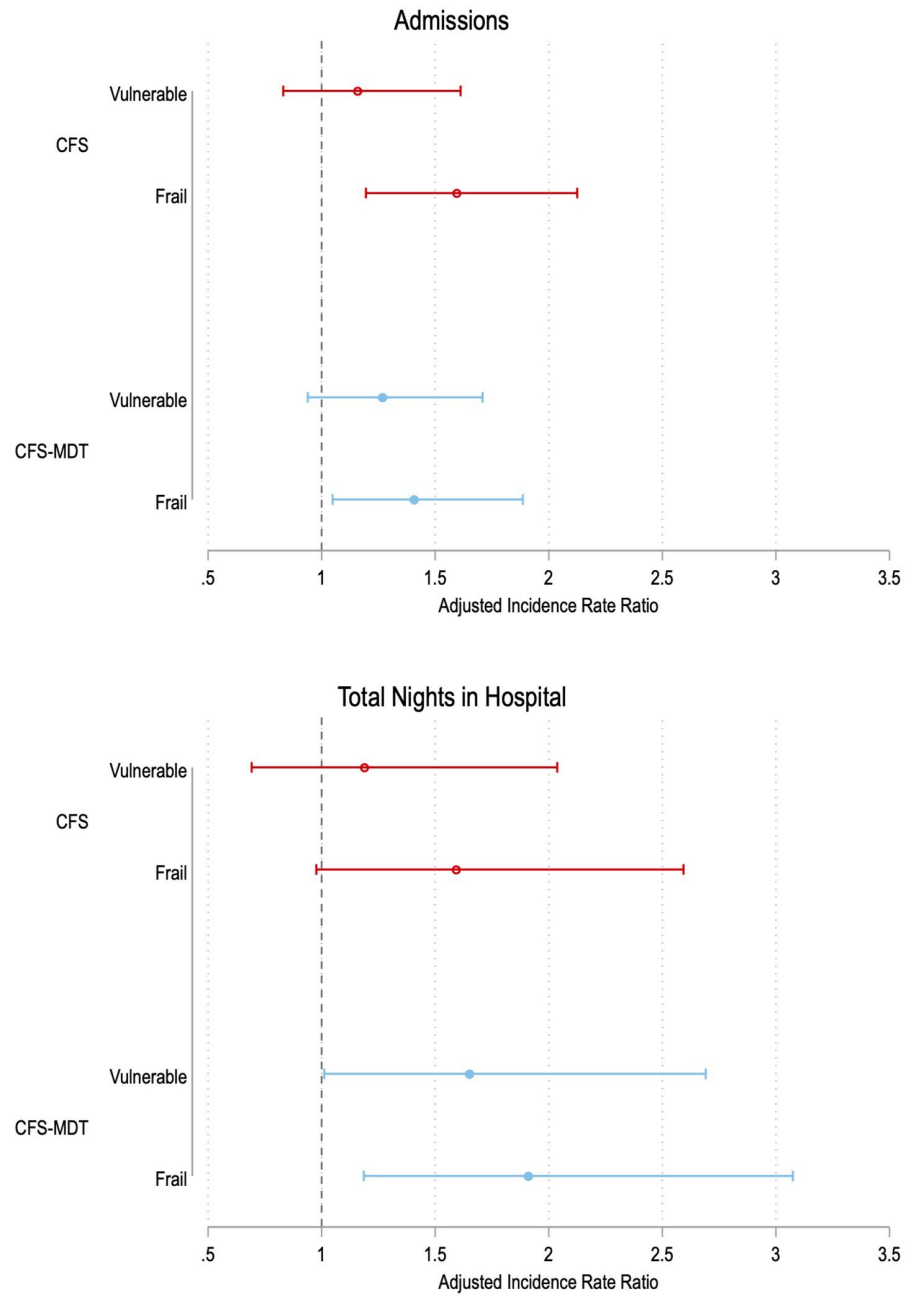
Forest plot of incidence rate ratios for admissions and total nights spent in hospital during follow-up by frailty category for CFS and CFS-MDT

Table 5 shows that increasing frailty was associated with higher rates of admission during follow up by both tools on adjusted analyses. Both tools demonstrated association with greater nights spent in hospital on univariable analysis, but only the CFS-MDT retained this association on multivariable analysis. Figure 5; Table 6 show a similar pattern was observed when the frailty tools were divided into frailty categories. Fully adjusted negative binomial models are shown in Supplementary tables S11-18.

**Table 5:** Incidence Rate Ratios of hospital admission, total nights in hospital by frailty as a continuous variable

	CFS		CFS-MDT	
	IRR	P	IRR	P
	95% C.I.		95% C.I.	
*Total Admissions*				
Univariable	**1.26**	**< 0.001**	**1.22**	**< 0.001**
	**1.17–1.36**		**1.14–1.30**	
Multivariable	**1.14**	**0.006**	**1.10**	**0.020**
	**1.04–1.25**		**1.02–1.19**	
*Total Nights in Hospital*				
Univariable	**1.34**	**< 0.001**	**1.36**	**< 0.001**
	**1.19–1.51**		**1.24–1.49**	
Multivariable	1.13	**0.126**	**1.22**	**0.001**
	0.97–1.33		**1.08–1.38**	

Incidence Rate Ratios and 95% confidence intervals shown. All values obtained by negative binomial regression except *, which was obtained using zero-truncated negative binomial regression

**Table 6:** Incidence Rate Ratios of hospital admission, total nights in hospital by frailty as an ordinal variable

		CFS		CFS-MDT	
		IRR	P	IRR	P
		95% C.I.		95% C.I.	
*Total Admissions*					
Univariable	Vulnerable	1.35	0.084	**1.51**	**0.006**
		0.96–1.91		**1.12–2.03**	
	Frail	**2.10**	**< 0.001**	**1.96**	**< 0.001**
		**1.61–2.73**		**1.51–2.53**	
Multivariable	Vulnerable	1.16	0.387	1.27	0.121
		0.83–1.61		0.94–1.71	
	Frail	**1.59**	**0.002**	**1.41**	**0.023**
		**1.20–2.13**		**1.05–1.89**	
*Total Nights in Hospital*					
Univariable	Vulnerable	1.52	0.111	1.56	0.052
		0.91–2.53		1.00-2.45	
	Frail	**2.71**	**< 0.001**	**2.85**	**< 0.001**
		**1.84–4.01**		**1.92–4.22**	
Multivariable	Vulnerable	1.19	0.533	1.52	0.090
		0.69–2.04		0.94–2.46	
	Frail	1.59	0.062	**1.75**	**0.013**
		0.97–2.59		**1.12–2.72**	

Incidence Rate Ratios and 95% confidence intervals shown. All values obtained by negative binomial regression

## Discussion

Frailty is associated with adverse clinical outcomes for haemodialysis patients, including increased risk for hospitalisation and/or mortality, but heterogeneity in application and reporting of frailty tools limits generalisability. The CFS has been studied in haemodialysis, but based upon clinician opinion rather than—as originally operationalised—an adjunct to detailed clinical interview [5]. In this large prospective single centre prevalent haemodialysis cohort, we have shown that CFS derived via MDT discussion yields a much lower frailty prevalence than that based upon responses to clinical interview. Furthermore, there was only weak correlation of raw CFS scores and minimal agreement upon frailty classification between the two approaches. Whilst those adjudged to be frail both CFS and CFS-MDT had greater hazard for mortality/hospitalisation than robust participants, only frailty by CFS retained this association on multivariable analysis. It should be noted that when treated as a continuous variable—as originally intended [4]—increasing frailty by both scores was associated with mortality/hospitalisation and rate of admissions on adjusted analyses. Conversely, only the CFS-MDT was associated with increased nights in hospital on multivariable analysis. These results prompt questions about the validity of using clinician opinion to guide CFS scoring without the initial clinical interview, and highlights difficulties comparing results between cohorts that employ different frailty methodologies.

The CFS was originally conceived as a simple measure of frailty, validated after clinical interview of subjects. In this context, it showed high correlation with other frailty scores [4]. It has subsequently been studied as a subjective measure in incident haemodialysis based upon the opinion of the primary nephrologist [5], although this measure has not been validated against the CFS as originally conceived. Nevertheless, increasing frailty by this approach was associated with higher mortality. We have previously shown that whilst agreement between the CFS and other validated frailty scores was weak or minimal, correlation of raw CFS scores with the Frailty Index was strong, and moderate correlation was seen between CFS and Frailty Phenotype and Edmonton Frail Scale [3].

Our findings of wide variation in frailty prevalence, alongside the weak correlation and minimal agreement between the CFS and CFS-MDT, raises concern for clinical application. Figure 2 shows that over half of participants adjudged frail and over three-quarters of those adjudged vulnerable were misclassified by the CFS-MDT. Indeed, the frailty prevalence of 26.5% by CFS-MDT is less than half that of the CFS and also substantially lower than prevalence estimates according to the Frailty Phenotype, Frailty Index, and Edmonton Frail Scale in this cohort.[3] This suggests that relying upon clinician judgement alone may not be sufficient to capture all frailty within haemodialysis, which risks leaving behind a significant proportion of potential candidates for frailty intervention. The significant variation between haemodialysis units in both correlation of raw frailty scores and in agreement upon frailty status between the CFS and CFS-MDT suggests unmeasured local factors at individual haemodialysis units may influence interpretation of CFS-MDT and raises questions about inter-rater reliability.

Importantly, both the CFS and CFS-MDT were associated with mortality, mortality/hospitalisation, and total number of hospital admissions after adjustment for important covariables. However, only the CFS-MDT retained significant association with nights spent in hospital upon adjusted analyses. This complicates the notion of the CFS-MDT as an ‘inferior’ frailty tool. The CFS relies upon a perhaps simplistic decoding of ADL disability, whereas the subjective CFS-MDT may result in a more nuanced reflection of patients’ clinical status by the clinical team who know them best. An alternative hypothesis, and one which may explain the lower overall rates of frailty using CFS-MDT, may be that clinicians are liable to compare patients against one another in a haemodialysis unit. This may have the dual effect of pushing down the estimate of frailty prevalence (as patient *x* is not as frail as patient *y*), but also of offering discrimination between those at higher and lower risk of adverse outcomes.

Frailty prevalence by the CFS-MDT in our prevalent haemodialysis cohort is remarkably similar to the 26% obtained from a Canadian incident haemodialysis cohort with a CFS obtained by similar methodology [5]. We must exercise caution when comparing frailty estimates across cohorts as demographics and methodologies may differ significantly. However, the discrepancy observed in the FITNESS cohort between frailty prevalence by CFS-MDT and CFS—alongside other frailty scores[3]—may lead us to speculate whether frailty prevalence might have been higher in the Canadian cohort if assessed by other frailty tools. Certainly, such a discrepancy between frailty prevalence observed between two interpretations of the same frailty instrument in the FITNESS cohort strongly suggests a need for consensus upon (1) which frailty tool is most appropriate for widespread use, and (2) the specific application of said tool. This would limit what may otherwise be unsatisfactory variation between methodologies and aid the kidney community in comparing the needs of different haemodialysis populations.

To our knowledge, FITNESS is the largest prospective cohort study to compare frequently used frailty tools in haemodialysis, and the first to compare their associations with both mortality and hospitalisation. Further strengths include diversity of demographics, comorbidities and socio-economic backgrounds representative of local demographics [18]. However our data should be interpreted with caution in non-English populations. A limitation of FITNESS is the single cross-sectional assessment, as frailty is a dynamic state, with year-by-year variability in frailty observed in a prospective US haemodialysis cohort [19]. The CFS was derived from investigator interrogation of ADL questionnaire responses, rather than via CGA followed by multi-disciplinary discussion as in the original validation cohort [4]. We would argue, however that the CFS here closely represents real-world application of the tool in clinical practice [13]. Our classifications of robust, vulnerable, and frail risks applying an arbitrary cut-off of frailty which may influence results. However, these were chosen in previous work to allow assessment of agreement across different frailty scores [3], and we may argue they hold close resemblance to the original score descriptors for the CFS. For example, a score of 4 is “vulnerable” by both our definition and the original CFS studies, whilst a score ≥ 5 comprises increasing degrees of frailty [13, 15]. It remains important to recognise—as we have previously argued—that frailty is best considered as a continuum rather than through imposition of cut-off levels [3]. In another limitation, some 95% confidence intervals crossed the point of no effect by small margins, raising the possibility of type II error. Finally, whilst these data describe associations with mortality and hospital admissions, they cannot support the notion of frailty as a ‘predictor’ of such adverse outcomes, and due caution should be exercised when considering the applicability of these findings to the individual haemodialysis recipient in clinical practice.

## Conclusions

To conclude, assessment of the Clinical Frailty Scale is dramatically affected by the underlying methodology, with the potential to profoundly affect clinical decision making. Agreement between subjective and objective measures of CFS frailty is minimal, with vulnerable patients at greatest risk of misclassification. Whilst the subjective CFS-MDT showed greater association with some adverse outcomes than the traditional CFS, the minimal agreement and weak correlation between the scores, alongside wide variation in the agreement and correlation between dialysis units suggests the subjective measure of CFS-MDT is a poor alternative to the validated score. Standardisation of CFS use is of paramount importance in clinical and research practice within haemodialysis cohorts so that the lessons of frailty may be more freely applicable across populations.

## Electronic supplementary material

Below is the link to the electronic supplementary material.

Supplementary Material 1

## Data Availability

The datasets used and/or analysed during the current study are available from the corresponding author on reasonable request.

## List of abbreviations

ADL: Activities of Daily Living
CFS: Clinical Frailty Scale
CFS-MDT: Clinical Frailty Scale derived from MDT discussion
CKD: Chronic Kidney Disease
C.I.: Confidence Interval
COVID-19: Coronavirus disease of 2019
EPR: Electronic Patient Record
FITNESS: Frailty Intervention Trial iN End-Stage patients on haemodialysiS
HES: Hospital Episode Statistics
HR: Hazard Ratio
IQR: Inter-quartile range
IMD: English Indices of Multiple Deprivation
IRR: Incidence Rate Ratio
MACE: Major Adverse Cardiovascular Events
MDT: Multi-disciplinary team
MoCA: Montreal Cognitive Assessment
NHS: National Health Service
ONS: Office for National Statistics
QA: Quality Assurance
