# Supplementary material for: A clinical frailty scale obtained from MDT discussion performs poorly in assessing frailty in haemodialysis recipients
Source: BMC Nephrol. 2023 Mar 30;24:80. doi: 10.1186/s12882-023-03126-0 (PMC10062243; doi:10.1186/s12882-023-03126-0)
Supplement: Supplementary file 1 — Supplementary Material 1 [file 12882_2023_3126_MOESM1_ESM.docx]

**Supplementary Table S1: Admission diagnosis group and constituent HES/OPCS-4 codes:**

HES= Hospital Episode Statistics OPCS-4=Office of Population Censuses and Surveys Classification of Interventions and Procedures.. MACE=Major Adverse Cardiovascular Events.

**Supplementary Table S2: Top 10 causes of death**

**Supplementary Table S3: Fully adjusted Cox Regression Model of mortality associated with CFS frailty status - ordinal variable**

|  | HR | Lower 95% C.I. | Upper 95% C.I. | P |
| --- | --- | --- | --- | --- |
| CFS |  |  |  |  |
| *Robust* | REFERENCE | | | |
| *Vulnerable* | 2.76 | 1.12 | 6.83 | 0.028 |
| *Frail* | 3.60 | 1.56 | 8.34 | 0.003 |
| Age | 0.99 | 0.97 | 1.02 | 0.635 |
| Gender |  |  |  |  |
| *Male* | REFERENCE | | | |
| *Female* | 0.89 | 0.58 | 1.38 | 0.608 |
| Ethnicity |  |  |  |  |
| *White* | REFERENCE | | | |
| *South Asian* | 0.68 | 0.37 | 1.25 | 0.214 |
| *Black* | 0.90 | 0.48 | 1.66 | 0.726 |
| *Other* | 0.85 | 0.11 | 6.59 | 0.88 |
| BMI | 0.99 | 0.96 | 1.02 | 0.408 |
| IMD Quintile |  |  |  |  |
| *1* | REFERENCE | | | |
| *2* | 0.90 | 0.50 | 1.64 | 0.729 |
| *3* | 1.15 | 0.64 | 2.06 | 0.644 |
| *4* | 0.83 | 0.36 | 1.92 | 0.670 |
| *5* | 0.76 | 0.31 | 1.88 | 0.560 |
| *Unknown* | 0.83 | 0.29 | 2.38 | 0.723 |
| Charlson Index* | 1.18 | 1.05 | 1.33 | 0.006 |
| Previous admissions | 1.03 | 0.93 | 1.14 | 0.562 |
| Number Medications | 1.00 | 0.93 | 1.07 | 0.948 |
| Smoking Status |  |  |  |  |
| *Current Smoker* | REFERENCE | | | |
| *Ex-Smoker* | 1.48 | 0.75 | 2.90 | 0.256 |
| *Never Smoked* | 0.89 | 0.46 | 1.73 | 0.728 |
| Albumin (g/L) | 0.94 | 0.90 | 0.98 | 0.005 |
| Walking Aid Use |  |  |  |  |
| *No* | REFERENCE | | | |
| *Yes* | 1.31 | 0.77 | 2.22 | 0.316 |
| HD vintage | 1.00 | 1.00 | 1.00 | 0.786 |
| Transplant Listing Status |  |  |  |  |
| *Not Active/Not Listed* | REFERENCE | | | |
| *Active* | 0.18 | 0.02 | 1.32 | 0.091 |

HR = Hazard Ratio. *=CKD omitted. **= Number of admissions in year prior to assessment.

**Supplementary Table S4: Fully adjusted Cox Regression Model of mortality associated with CFS-MDT frailty status - ordinal variable**

|  | HR | Lower 95% C.I. | Upper 95% C.I. | P |
| --- | --- | --- | --- | --- |
| CFS-MDT |  |  |  |  |
| *Robust* | REFERENCE | | | |
| *Vulnerable* | 2.03 | 1.10 | 3.77 | 0.024 |
| *Frail* | 2.30 | 1.30 | 4.07 | 0.004 |
| Age | 0.99 | 0.97 | 1.02 | 0.661 |
| Gender |  |  |  |  |
| *Male* | REFERENCE | | | |
| *Female* | 0.87 | 0.56 | 1.36 | 0.542 |
| Ethnicity |  |  |  |  |
| *White* | REFERENCE | | | |
| *South Asian* | 0.67 | 0.36 | 1.23 | 0.194 |
| *Black* | 0.90 | 0.49 | 1.67 | 0.746 |
| *Other* | 0.85 | 0.11 | 6.58 | 0.878 |
| BMI | 0.99 | 0.96 | 1.02 | 0.402 |
| IMD Quintile |  |  |  |  |
| *1* | REFERENCE | | | |
| *2* | 0.85 | 0.47 | 1.55 | 0.604 |
| *3* | 1.04 | 0.58 | 1.88 | 0.884 |
| *4* | 0.77 | 0.33 | 1.77 | 0.534 |
| *5* | 0.73 | 0.30 | 1.76 | 0.478 |
| *Unknown* | 0.79 | 0.27 | 2.26 | 0.659 |
| Charlson Index* | 1.16 | 1.03 | 1.31 | 0.015 |
| Previous admissions | 1.04 | 0.94 | 1.15 | 0.415 |
| Number Medications | 1.01 | 0.94 | 1.07 | 0.878 |
| Smoking Status |  |  |  |  |
| *Current Smoker* | REFERENCE | | | |
| *Ex-Smoker* | 1.38 | 0.70 | 2.72 | 0.346 |
| *Never Smoked* | 0.84 | 0.43 | 1.61 | 0.592 |
| Albumin (g/L) | 0.94 | 0.90 | 0.98 | 0.008 |
| Walking Aid Use |  |  |  |  |
| *No* | REFERENCE | | | |
| *Yes* | 1.35 | 0.79 | 2.32 | 0.276 |
| HD vintage | 1.00 | 1.00 | 1.00 | 0.583 |
| Transplant Listing Status |  |  |  |  |
| *Not Active/Not Listed* | REFERENCE | | | |
| *Active* | 0.20 | 0.03 | 1.49 | 0.116 |

HR = Hazard Ratio. *=CKD omitted. **= Number of admissions in year prior to assessment.

**Supplementary Table S5: Fully adjusted Cox Regression Model of mortality associated with CFS as continuous variable**

|  | HR | Lower 95% C.I. | Upper 95% C.I. | P |
| --- | --- | --- | --- | --- |
| CFS | 1.39 | 1.13 | 1.70 | 0.002 |
| Age | 1.00 | 0.97 | 1.02 | 0.761 |
| Gender |  |  |  |  |
| *Male* | 1.00 |  |  |  |
| *Female* | 0.87 | 0.56 | 1.36 | 0.550 |
| Ethnicity |  |  |  |  |
| *White* | 1.00 |  |  |  |
| *South Asian* | 0.68 | 0.37 | 1.24 | 0.207 |
| *Black* | 0.94 | 0.51 | 1.74 | 0.845 |
| *Other* | 0.69 | 0.09 | 5.29 | 0.721 |
| BMI | 0.98 | 0.96 | 1.01 | 0.304 |
| IMD Quintile |  |  |  |  |
| *1* | 1.00 |  |  |  |
| *2* | 0.94 | 0.52 | 1.70 | 0.830 |
| *3* | 1.15 | 0.64 | 2.06 | 0.647 |
| *4* | 0.86 | 0.37 | 1.98 | 0.728 |
| *5* | 0.79 | 0.32 | 1.95 | 0.616 |
| *Unknown* | 0.82 | 0.29 | 2.36 | 0.718 |
| Charlson Index* | 1.17 | 1.04 | 1.32 | 0.010 |
| Previous admissions | 1.03 | 0.93 | 1.13 | 0.624 |
| Number Medications | 1.00 | 0.93 | 1.06 | 0.898 |
| Smoking Status |  |  |  |  |
| *Current Smoker* | 1.00 |  |  |  |
| *Ex-Smoker* | 1.47 | 0.75 | 2.89 | 0.264 |
| *Never Smoked* | 0.86 | 0.44 | 1.67 | 0.660 |
| Albumin (g/L) | 0.94 | 0.90 | 0.98 | 0.004 |
| Walking Aid Use |  |  |  |  |
| *No* | 1.00 |  |  |  |
| *Yes* | 1.21 | 0.70 | 2.11 | 0.491 |
| HD vintage | 1.00 | 1.00 | 1.00 | 0.776 |
| Transplant Listing Status |  |  |  |  |
| *Not Active/Not Listed* | 1.00 |  |  |  |
| *Active* | 0.17 | 0.02 | 1.27 | 0.084 |

HR = Hazard Ratio. *=CKD omitted. **= Number of admissions in year prior to assessment.

**Supplementary Table S6: Fully adjusted Cox Regression Model of mortality associated with CFS-MDT as continuous variable**

|  | HR | Lower 95% C.I. | Upper 95% C.I. | P |
| --- | --- | --- | --- | --- |
| CFS-MDT | 1.36 | 1.16 | 1.59 | <0.001 |
| Age | 1.00 | 0.97 | 1.02 | 0.802 |
| Gender |  |  |  |  |
| *Male* | REFERENCE | | | |
| *Female* | 0.85 | 0.55 | 1.32 | 0.471 |
| Ethnicity |  |  |  |  |
| *White* | REFERENCE | | | |
| *South Asian* | 0.68 | 0.37 | 1.25 | 0.213 |
| *Black* | 0.92 | 0.50 | 1.69 | 0.782 |
| *Other* | 1.00 | 0.13 | 7.68 | 0.996 |
| BMI | 0.99 | 0.96 | 1.01 | 0.327 |
| IMD Quintile |  |  |  |  |
| *1* | REFERENCE | | | |
| *2* | 0.92 | 0.51 | 1.66 | 0.781 |
| *3* | 1.11 | 0.62 | 2.01 | 0.717 |
| *4* | 0.80 | 0.35 | 1.84 | 0.597 |
| *5* | 0.79 | 0.33 | 1.92 | 0.607 |
| *Unknown* | 0.79 | 0.27 | 2.26 | 0.655 |
| Charlson Index* | 1.15 | 1.02 | 1.30 | 0.027 |
| Previous admissions | 1.04 | 0.94 | 1.15 | 0.443 |
| Number Medications | 1.00 | 0.94 | 1.07 | 0.903 |
| Smoking Status |  |  |  |  |
| *Current Smoker* | REFERENCE | | | |
| *Ex-Smoker* | 1.44 | 0.73 | 2.82 | 0.293 |
| *Never Smoked* | 0.82 | 0.43 | 1.58 | 0.554 |
| Albumin (g/L) | 0.95 | 0.91 | 1.00 | 0.036 |
| Walking Aid Use |  |  |  |  |
| *No* | REFERENCE | | | |
| *Yes* | 1.25 | 0.72 | 2.16 | 0.435 |
| HD vintage | 1.00 | 1.00 | 1.00 | 0.850 |
| Transplant Listing Status |  |  |  |  |
| *Not Active/Not Listed* | REFERENCE | | | |
| *Active* | 0.22 | 0.03 | 1.64 | 0.139 |

HR = Hazard Ratio. *=CKD omitted. **= Number of admissions in year prior to assessment.

**Supplementary Table S7: Fully adjusted Cox Regression Model of mortality/hospitalisation associated with CFS status - ordinal variable**

|  | HR | Lower 95% C.I. | Upper 95% C.I. | P |
| --- | --- | --- | --- | --- |
| CFS |  |  |  |  |
| *Robust* | REFERENCE | | | |
| *Vulnerable* | 1.22 | 0.85 | 1.75 | 0.281 |
| *Frail* | 1.56 | 1.13 | 2.16 | 0.008 |
| Age | 0.99 | 0.98 | 1.01 | 0.295 |
| Gender |  |  |  |  |
| *Male* | REFERENCE | | | |
| *Female* | 0.93 | 0.74 | 1.18 | 0.549 |
| Ethnicity |  |  |  |  |
| *White* | REFERENCE | | | |
| *South Asian* | 1.09 | 0.81 | 1.46 | 0.567 |
| *Black* | 1.00 | 0.72 | 1.40 | 0.991 |
| *Other* | 0.74 | 0.34 | 1.61 | 0.444 |
| BMI | 0.99 | 0.97 | 1.01 | 0.234 |
| IMD Quintile |  |  |  |  |
| *1* | REFERENCE | | | |
| *2* | 1.01 | 0.74 | 1.38 | 0.967 |
| *3* | 0.75 | 0.53 | 1.05 | 0.091 |
| *4* | 0.91 | 0.59 | 1.39 | 0.653 |
| *5* | 0.63 | 0.39 | 1.03 | 0.067 |
| *Unknown* | 0.89 | 0.55 | 1.42 | 0.624 |
| Charlson Index* | 1.03 | 0.96 | 1.11 | 0.398 |
| Previous admissions | 1.09 | 1.04 | 1.15 | 0.000 |
| Number Medications | 1.04 | 1.00 | 1.07 | 0.054 |
| Smoking Status |  |  |  |  |
| *Current Smoker* | REFERENCE | | | |
| *Ex-Smoker* | 0.86 | 0.60 | 1.24 | 0.419 |
| *Never Smoked* | 0.86 | 0.61 | 1.22 | 0.397 |
| Albumin (g/L) | 0.97 | 0.94 | 0.99 | 0.006 |
| Walking Aid Use |  |  |  |  |
| *No* | REFERENCE | | | |
| *Yes* | 1.12 | 0.85 | 1.48 | 0.425 |
| HD vintage | 1.00 | 1.00 | 1.00 | 0.201 |
| Transplant Listing Status |  |  |  |  |
| *Not Active/Not Listed* | REFERENCE | | | |
| *Active* | 0.93 | 0.63 | 1.36 | 0.695 |

HR = Hazard Ratio. *=CKD omitted. **= Number of admissions in year prior to assessment.

**Supplementary Table S8: Fully adjusted Cox Regression Model of mortality/hospitalisation associated with CFS-MDT status - ordinal variable**

|  | HR | Lower 95% C.I. | Upper 95% C.I. | P |
| --- | --- | --- | --- | --- |
| CFS-MDT |  |  |  |  |
| *Robust* | 1.00 |  |  |  |
| *Vulnerable* | 1.08 | 0.79 | 1.48 | 0.634 |
| *Frail* | 1.35 | 0.99 | 1.85 | 0.058 |
| Age | 0.99 | 0.98 | 1.01 | 0.363 |
| Gender |  |  |  |  |
| *Male* | 1.00 |  |  |  |
| *Female* | 0.94 | 0.74 | 1.19 | 0.608 |
| Ethnicity |  |  |  |  |
| *White* | 1.00 |  |  |  |
| *South Asian* | 1.12 | 0.84 | 1.51 | 0.431 |
| *Black* | 1.04 | 0.75 | 1.46 | 0.797 |
| *Other* | 0.73 | 0.34 | 1.60 | 0.438 |
| BMI | 0.99 | 0.97 | 1.01 | 0.181 |
| IMD Quintile |  |  |  |  |
| *1* | 1.00 |  |  |  |
| *2* | 1.00 | 0.73 | 1.37 | 0.993 |
| *3* | 0.74 | 0.53 | 1.03 | 0.078 |
| *4* | 0.91 | 0.59 | 1.39 | 0.653 |
| *5* | 0.61 | 0.38 | 1.00 | 0.050 |
| *Unknown* | 0.88 | 0.55 | 1.41 | 0.590 |
| Charlson Index* | 1.02 | 0.95 | 1.10 | 0.566 |
| Previous admissions | 1.09 | 1.04 | 1.14 | 0.000 |
| Number Medications | 1.04 | 1.00 | 1.08 | 0.030 |
| Smoking Status |  |  |  |  |
| *Current Smoker* | 1.00 |  |  |  |
| *Ex-Smoker* | 0.87 | 0.61 | 1.26 | 0.470 |
| *Never Smoked* | 0.87 | 0.61 | 1.23 | 0.429 |
| Albumin (g/L) | 0.97 | 0.94 | 0.99 | 0.009 |
| Walking Aid Use |  |  |  |  |
| *No* | 1.00 |  |  |  |
| *Yes* | 1.24 | 0.95 | 1.61 | 0.113 |
| HD vintage | 1.00 | 1.00 | 1.00 | 0.215 |
| Transplant Listing Status |  |  |  |  |
| *Not Active/Not Listed* | 1.00 |  |  |  |
| *Active* | 0.96 | 0.65 | 1.42 | 0.851 |

HR = Hazard Ratio. *=CKD omitted. **= Number of admissions in year prior to assessment.

**Supplementary Table S9: Fully adjusted Cox Regression Model of mortality/hospitalisation associated with CFS – continuous variable**

|  | HR | Lower 95% C.I. | Upper 95% C.I. | P |
| --- | --- | --- | --- | --- |
| CFS | 1.14 | 1.03 | 1.26 | 0.010 |
| Age | 0.99 | 0.98 | 1.00 | 0.251 |
| Gender |  |  |  |  |
| *Male* | 1.00 |  |  |  |
| *Female* | 0.92 | 0.73 | 1.17 | 0.501 |
| Ethnicity |  |  |  |  |
| *White* | 1.00 |  |  |  |
| *South Asian* | 1.10 | 0.82 | 1.48 | 0.507 |
| *Black* | 1.02 | 0.73 | 1.42 | 0.910 |
| *Other* | 0.73 | 0.33 | 1.59 | 0.424 |
| BMI | 0.99 | 0.97 | 1.01 | 0.207 |
| IMD Quintile |  |  |  |  |
| *1* | 1.00 |  |  |  |
| *2* | 1.04 | 0.76 | 1.42 | 0.805 |
| *3* | 0.77 | 0.55 | 1.07 | 0.121 |
| *4* | 0.94 | 0.61 | 1.45 | 0.787 |
| *5* | 0.64 | 0.39 | 1.05 | 0.078 |
| *Unknown* | 0.90 | 0.56 | 1.43 | 0.649 |
| Charlson Index* | 1.03 | 0.96 | 1.11 | 0.380 |
| Previous admissions** | 1.09 | 1.04 | 1.14 | <0.001 |
| Number Medications | 1.03 | 1.00 | 1.07 | 0.057 |
| Smoking Status |  |  |  |  |
| *Current Smoker* | 1.00 |  |  |  |
| *Ex-Smoker* | 0.87 | 0.61 | 1.25 | 0.454 |
| *Never Smoked* | 0.86 | 0.61 | 1.22 | 0.403 |
| Albumin (g/L) | 0.97 | 0.94 | 0.99 | 0.006 |
| Walking Aid Use |  |  |  |  |
| *No* | 1.00 |  |  |  |
| *Yes* | 1.12 | 0.85 | 1.48 | 0.422 |
| HD vintage | 1.00 | 1.00 | 1.00 | 0.214 |
| Transplant Listing Status |  |  |  |  |
| *Not Active/Not Listed* | 1.00 |  |  |  |
| *Active* | 0.93 | 0.64 | 1.36 | 0.711 |

HR = Hazard Ratio. *=CKD omitted. **= Number of admissions in year prior to assessment.

**Supplementary Table S10: Fully adjusted Cox Regression Model of mortality/hospitalisation associated with CFS-MDT – continuous variable**

|  | HR | Lower 95% C.I. | Upper 95% C.I. | P |
| --- | --- | --- | --- | --- |
| CFS-MDT | 1.12 | 1.02 | 1.23 | 0.019 |
| Age | 0.99 | 0.98 | 1.01 | 0.382 |
| Gender |  |  |  |  |
| *Male* | 1.00 |  |  |  |
| *Female* | 0.93 | 0.74 | 1.18 | 0.552 |
| Ethnicity |  |  |  |  |
| *White* | 1.00 |  |  |  |
| *South Asian* | 1.12 | 0.84 | 1.50 | 0.442 |
| *Black* | 1.04 | 0.74 | 1.45 | 0.829 |
| *Other* | 0.73 | 0.34 | 1.60 | 0.432 |
| BMI | 0.99 | 0.97 | 1.01 | 0.182 |
| IMD Quintile |  |  |  |  |
| *1* | 1.00 |  |  |  |
| *2* | 1.01 | 0.74 | 1.39 | 0.934 |
| *3* | 0.76 | 0.54 | 1.06 | 0.105 |
| *4* | 0.92 | 0.60 | 1.40 | 0.690 |
| *5* | 0.65 | 0.40 | 1.06 | 0.084 |
| *Unknown* | 0.89 | 0.56 | 1.43 | 0.636 |
| Charlson Index* | 1.02 | 0.95 | 1.10 | 0.583 |
| Previous admissions** | 1.09 | 1.04 | 1.14 | <0.001 |
| Number Medications | 1.04 | 1.00 | 1.07 | 0.039 |
| Smoking Status |  |  |  |  |
| *Current Smoker* | 1.00 |  |  |  |
| *Ex-Smoker* | 0.87 | 0.61 | 1.26 | 0.466 |
| *Never Smoked* | 0.86 | 0.61 | 1.21 | 0.382 |
| Albumin (g/L) | 0.97 | 0.95 | 1.00 | 0.023 |
| Walking Aid Use |  |  |  |  |
| *No* | 1.00 |  |  |  |
| *Yes* | 1.20 | 0.92 | 1.57 | 0.174 |
| HD vintage | 1.00 | 1.00 | 1.00 | 0.226 |
| Transplant Listing Status |  |  |  |  |
| *Not Active/Not Listed* | 1.00 |  |  |  |
| *Active* | 1.00 | 0.68 | 1.48 | 0.999 |

HR = Hazard Ratio. *=CKD omitted. **= Number of admissions in year prior to assessment.

**Supplementary Table S11: Fully adjusted negative binomial regression of hospital admissions associated with CFS – continuous variable**

|  | IRR | Lower 95% C.I. | Upper 95% C.I. | P |
| --- | --- | --- | --- | --- |
| CFS | 1.14 | 1.04 | 1.25 | 0.006 |
| Age | 0.99 | 0.98 | 1.00 | 0.047 |
| Gender |  |  |  |  |
| *Male* | 1.00 |  |  |  |
| *Female* | 0.96 | 0.78 | 1.20 | 0.742 |
| Ethnicity |  |  |  |  |
| *White* | 1.00 |  |  |  |
| *South Asian* | 0.79 | 0.59 | 1.05 | 0.109 |
| *Black* | 0.65 | 0.47 | 0.90 | 0.008 |
| *Other* | 0.85 | 0.41 | 1.76 | 0.667 |
| BMI | 0.99 | 0.98 | 1.01 | 0.267 |
| IMD Quintile |  |  |  |  |
| *1* | 1.00 |  |  |  |
| *2* | 0.88 | 0.65 | 1.18 | 0.383 |
| *3* | 0.75 | 0.55 | 1.03 | 0.074 |
| *4* | 0.83 | 0.56 | 1.25 | 0.375 |
| *5* | 0.71 | 0.45 | 1.12 | 0.142 |
| *Unknown* | 1.19 | 0.78 | 1.82 | 0.408 |
| Charlson Index* | 1.09 | 1.02 | 1.17 | 0.011 |
| Previous admissions** | 1.09 | 1.04 | 1.15 | <0.001 |
| Number Medications | 1.03 | 1.00 | 1.06 | 0.087 |
| Smoking Status |  |  |  |  |
| *Current Smoker* | 1.00 |  |  |  |
| *Ex-Smoker* | 1.02 | 0.71 | 1.45 | 0.933 |
| *Never Smoked* | 0.89 | 0.65 | 1.23 | 0.491 |
| Albumin (g/L) | 0.98 | 0.96 | 1.01 | 0.157 |
| Walking Aid Use |  |  |  |  |
| *No* | 1.00 |  |  |  |
| *Yes* | 1.37 | 1.06 | 1.78 | 0.017 |
| HD vintage | 1.00 | 1.00 | 1.00 | 0.566 |
| Transplant Listing Status |  |  |  |  |
| *Not Active/Not Listed* | 1.00 |  |  |  |
| *Active* | 0.85 | 0.59 | 1.23 | 0.390 |
| Constant | 0.01 | 0.00 | 0.02 | <0.001 |

IRR= Incidence Rate Ratio, obtained by negative binomial regression. *=CKD omitted. **= Number of admissions in year prior to assessment.

**Supplementary Table S12 Fully adjusted negative binomial regression of hospital admissions associated with CFS-MDT – continuous variable**

|  | IRR | Lower 95% C.I. | Upper 95% C.I. | P |
| --- | --- | --- | --- | --- |
| CFS-MDT | 1.10 | 1.02 | 1.19 | 0.020 |
| Age | 0.99 | 0.98 | 1.00 | 0.030 |
| Gender |  |  |  |  |
| *Male* | 1.00 |  |  |  |
| *Female* | 0.97 | 0.78 | 1.21 | 0.792 |
| Ethnicity |  |  |  |  |
| *White* | 1.00 |  |  |  |
| *South Asian* | 0.79 | 0.59 | 1.07 | 0.124 |
| *Black* | 0.65 | 0.47 | 0.89 | 0.008 |
| *Other* | 0.93 | 0.45 | 1.92 | 0.844 |
| BMI | 0.99 | 0.98 | 1.01 | 0.347 |
| IMD Quintile |  |  |  |  |
| *1* | 1.00 |  |  |  |
| *2* | 0.87 | 0.65 | 1.18 | 0.376 |
| *3* | 0.74 | 0.54 | 1.02 | 0.068 |
| *4* | 0.81 | 0.54 | 1.21 | 0.295 |
| *5* | 0.70 | 0.44 | 1.12 | 0.137 |
| *Unknown* | 1.17 | 0.76 | 1.78 | 0.478 |
| Charlson Index* | 1.09 | 1.02 | 1.17 | 0.014 |
| Previous admissions** | 1.09 | 1.04 | 1.14 | 0.001 |
| Number Medications | 1.03 | 1.00 | 1.07 | 0.046 |
| Smoking Status |  |  |  |  |
| *Current Smoker* | 1.00 |  |  |  |
| *Ex-Smoker* | 1.02 | 0.71 | 1.46 | 0.916 |
| *Never Smoked* | 0.90 | 0.65 | 1.25 | 0.538 |
| Albumin (g/L) | 0.99 | 0.97 | 1.01 | 0.501 |
| Walking Aid Use |  |  |  |  |
| *No* | 1.00 |  |  |  |
| *Yes* | 1.47 | 1.15 | 1.88 | 0.002 |
| HD vintage | 1.00 | 1.00 | 1.00 | 0.419 |
| Transplant Listing Status |  |  |  |  |
| *Not Active/Not Listed* | 1.00 |  |  |  |
| *Active* | 0.89 | 0.62 | 1.29 | 0.532 |
| Constant | 0.00 | 0.00 | 0.02 | <0.001 |

IRR= Incidence Rate Ratio, obtained by negative binomial regression. *=CKD omitted. **= Number of admissions in year prior to assessment.

**Supplementary Table S13: Fully adjusted negative binomial regression of total nights in hospital associated with CFS – continuous variable**

|  | IRR | Lower 95% C.I. | Upper 95% C.I. | P |
| --- | --- | --- | --- | --- |
| CFS | 1.13 | 0.97 | 1.33 | 0.126 |
| Age | 0.98 | 0.96 | 1.00 | 0.036 |
| Gender |  |  |  |  |
| *Male* | 1.00 |  |  |  |
| *Female* | 0.98 | 0.69 | 1.38 | 0.888 |
| Ethnicity |  |  |  |  |
| *White* | 1.00 |  |  |  |
| *South Asian* | 0.74 | 0.44 | 1.23 | 0.243 |
| *Black* | 0.76 | 0.45 | 1.29 | 0.310 |
| *Other* | 0.54 | 0.17 | 1.71 | 0.298 |
| BMI | 0.99 | 0.96 | 1.01 | 0.251 |
| IMD Quintile |  |  |  |  |
| *1* | 1.00 |  |  |  |
| *2* | 0.59 | 0.36 | 0.97 | 0.036 |
| *3* | 0.76 | 0.44 | 1.29 | 0.301 |
| *4* | 0.83 | 0.43 | 1.61 | 0.584 |
| *5* | 1.07 | 0.51 | 2.25 | 0.851 |
| *Unknown* | 1.14 | 0.57 | 2.28 | 0.714 |
| Charlson Index* | 1.11 | 0.99 | 1.25 | 0.086 |
| Previous admissions** | 1.13 | 1.03 | 1.22 | 0.007 |
| Number Medications | 1.00 | 0.95 | 1.06 | 0.882 |
| Smoking Status |  |  |  |  |
| *Current Smoker* | 1.00 |  |  |  |
| *Ex-Smoker* | 1.98 | 1.10 | 3.57 | 0.023 |
| *Never Smoked* | 1.29 | 0.76 | 2.19 | 0.353 |
| Albumin (g/L) | 0.96 | 0.92 | 0.99 | 0.019 |
| Walking Aid Use |  |  |  |  |
| *No* | 1.00 |  |  |  |
| *Yes* | 2.12 | 1.37 | 3.28 | 0.001 |
| HD vintage | 1.00 | 1.00 | 1.00 | 0.589 |
| Transplant Listing Status |  |  |  |  |
| *Not Active/Not Listed* | 1.00 |  |  |  |
| *Active* | 0.81 | 0.45 | 1.43 | 0.463 |
| Constant | 0.14 | 0.02 | 0.92 | 0.040 |

IRR= Incidence Rate Ratio, obtained by negative binomial regression. *=CKD omitted. **= Number of admissions in year prior to assessment.

**Supplementary Table S14: Fully adjusted negative binomial regression of total nights in hospital associated with CFS-MDT – continuous variable**

|  | IRR | Lower 95% C.I. | Upper 95% C.I. | P |
| --- | --- | --- | --- | --- |
| CFS-MDT | 1.22 | 1.08 | 1.38 | 0.001 |
| Age | 0.98 | 0.96 | 1.00 | 0.029 |
| Gender |  |  |  |  |
| *Male* | 1.00 |  |  |  |
| *Female* | 0.89 | 0.63 | 1.27 | 0.531 |
| Ethnicity |  |  |  |  |
| *White* | 1.00 |  |  |  |
| *South Asian* | 0.77 | 0.47 | 1.28 | 0.314 |
| *Black* | 0.74 | 0.44 | 1.25 | 0.261 |
| *Other* | 0.63 | 0.20 | 1.96 | 0.423 |
| BMI | 0.99 | 0.97 | 1.02 | 0.493 |
| IMD Quintile |  |  |  |  |
| *1* | 1.00 |  |  |  |
| *2* | 0.64 | 0.39 | 1.04 | 0.073 |
| *3* | 0.71 | 0.42 | 1.21 | 0.212 |
| *4* | 0.79 | 0.41 | 1.52 | 0.484 |
| *5* | 1.18 | 0.57 | 2.45 | 0.658 |
| *Unknown* | 1.07 | 0.53 | 2.13 | 0.855 |
| Charlson Index* | 1.08 | 0.97 | 1.22 | 0.170 |
| Previous admissions** | 1.12 | 1.03 | 1.22 | 0.008 |
| Number Medications | 1.00 | 0.95 | 1.05 | 0.992 |
| Smoking Status |  |  |  |  |
| *Current Smoker* | 1.00 |  |  |  |
| *Ex-Smoker* | 1.84 | 1.03 | 3.30 | 0.039 |
| *Never Smoked* | 1.21 | 0.71 | 2.06 | 0.478 |
| Albumin (g/L) | 0.98 | 0.95 | 1.02 | 0.355 |
| Walking Aid Use |  |  |  |  |
| *No* | 1.00 |  |  |  |
| *Yes* | 2.13 | 1.45 | 3.12 | <0.001 |
| HD vintage | 1.00 | 1.00 | 1.00 | 0.882 |
| Transplant Listing Status |  |  |  |  |
| *Not Active/Not Listed* | 1.00 |  |  |  |
| *Active* | 0.90 | 0.51 | 1.58 | 0.707 |
| Constant | 0.05 | 0.01 | 0.39 | 0.004 |

IRR= Incidence Rate Ratio, obtained by negative binomial regression. *=CKD omitted. **= Number of admissions in year prior to assessment.

**Supplementary Table S15: Fully adjusted negative binomial regression admissions associated with CFS frailty status – ordinal variable**

|  | IRR | Lower 95% C.I. | Upper 95% C.I. | P |
| --- | --- | --- | --- | --- |
| CFS |  |  |  |  |
| *Robust* | 1.00 |  |  |  |
| *Vulnerable* | 1.16 | 0.83 | 1.61 | 0.387 |
| *Frail* | 1.59 | 1.20 | 2.13 | 0.002 |
| Age | 0.99 | 0.98 | 1.00 | 0.044 |
| Gender |  |  |  |  |
| *Male* | 1.00 |  |  |  |
| *Female* | 0.96 | 0.78 | 1.20 | 0.736 |
| Ethnicity |  |  |  |  |
| *White* | 1.00 |  |  |  |
| *South Asian* | 0.78 | 0.58 | 1.05 | 0.098 |
| *Black* | 0.64 | 0.46 | 0.88 | 0.006 |
| *Other* | 0.84 | 0.41 | 1.75 | 0.648 |
| BMI | 0.99 | 0.98 | 1.01 | 0.270 |
| IMD Quintile |  |  |  |  |
| *1* | 1.00 |  |  |  |
| *2* | 0.87 | 0.64 | 1.17 | 0.353 |
| *3* | 0.73 | 0.53 | 1.00 | 0.047 |
| *4* | 0.81 | 0.54 | 1.21 | 0.309 |
| *5* | 0.66 | 0.42 | 1.05 | 0.080 |
| *Unknown* | 1.21 | 0.80 | 1.84 | 0.371 |
| Charlson Index* | 1.09 | 1.02 | 1.17 | 0.011 |
| Previous admissions** | 1.09 | 1.04 | 1.15 | <0.001 |
| Number Medications | 1.03 | 0.99 | 1.06 | 0.117 |
| Smoking Status |  |  |  |  |
| *Current Smoker* | 1.00 |  |  |  |
| *Ex-Smoker* | 1.02 | 0.71 | 1.46 | 0.913 |
| *Never Smoked* | 0.90 | 0.65 | 1.24 | 0.503 |
| Albumin (g/L) | 0.98 | 0.96 | 1.01 | 0.133 |
| Walking Aid Use |  |  |  |  |
| *No* | 1.00 |  |  |  |
| *Yes* | 1.36 | 1.06 | 1.76 | 0.017 |
| HD vintage | 1.00 | 1.00 | 1.00 | 0.527 |
| Transplant Listing Status |  |  |  |  |
| *Not Active/Not Listed* | 1.00 |  |  |  |
| *Active* | 0.84 | 0.59 | 1.21 | 0.355 |
| Constant | 0.01 | 0.00 | 0.02 | <0.001 |

IRR= Incidence Rate Ratio, obtained by negative binomial regression. *=CKD omitted. **= Number of admissions in year prior to assessment.

**Supplementary Table S16: Fully adjusted negative binomial regression admissions associated with CFS-MDT frailty status – ordinal variable**

|  | IRR | Lower 95% C.I. | Upper 95% C.I. | P |
| --- | --- | --- | --- | --- |
| CFS-MDT |  |  |  |  |
| *Robust* | 1.00 |  |  |  |
| *Vulnerable* | 1.27 | 0.94 | 1.71 | 0.121 |
| *Frail* | 1.41 | 1.05 | 1.89 | 0.023 |
| Age | 0.99 | 0.98 | 1.00 | 0.030 |
| Gender |  |  |  |  |
| *Male* | 1.00 |  |  |  |
| *Female* | 0.97 | 0.78 | 1.21 | 0.816 |
| Ethnicity |  |  |  |  |
| *White* | 1.00 |  |  |  |
| *South Asian* | 0.80 | 0.60 | 1.07 | 0.130 |
| *Black* | 0.65 | 0.47 | 0.89 | 0.008 |
| *Other* | 0.90 | 0.44 | 1.87 | 0.783 |
| BMI | 0.99 | 0.98 | 1.01 | 0.307 |
| IMD Quintile |  |  |  |  |
| *1* | 1.00 |  |  |  |
| *2* | 0.85 | 0.63 | 1.15 | 0.303 |
| *3* | 0.73 | 0.53 | 1.00 | 0.050 |
| *4* | 0.80 | 0.53 | 1.19 | 0.266 |
| *5* | 0.69 | 0.43 | 1.10 | 0.115 |
| *Unknown* | 1.17 | 0.77 | 1.79 | 0.467 |
| Charlson Index* | 1.09 | 1.02 | 1.17 | 0.013 |
| Previous admissions** | 1.09 | 1.04 | 1.15 | <0.001 |
| Number Medications | 1.03 | 1.00 | 1.07 | 0.049 |
| Smoking Status |  |  |  |  |
| *Current Smoker* | 1.00 |  |  |  |
| *Ex-Smoker* | 1.01 | 0.71 | 1.45 | 0.938 |
| *Never Smoked* | 0.90 | 0.65 | 1.25 | 0.543 |
| Albumin (g/L) | 0.99 | 0.97 | 1.01 | 0.371 |
| Walking Aid Use |  |  |  |  |
| *No* | 1.00 |  |  |  |
| *Yes* | 1.47 | 1.15 | 1.88 | 0.002 |
| HD vintage | 1.00 | 1.00 | 1.00 | 0.463 |
| Transplant Listing Status |  |  |  |  |
| *Not Active/Not Listed* | 1.00 |  |  |  |
| *Active* | 0.89 | 0.61 | 1.28 | 0.525 |
| Constant | 0.01 | 0.00 | 0.02 | <0.001 |

IRR= Incidence Rate Ratio, obtained by negative binomial regression. *=CKD omitted. **= Number of admissions in year prior to assessment.

**Supplementary Table S17: Fully adjusted negative binomial regression of total nights in hospital associated with CFS frailty status – ordinal variable**

|  | IRR | Lower 95% C.I. | Upper 95% C.I. | P |
| --- | --- | --- | --- | --- |
| CFS |  |  |  |  |
| *Robust* | 1.00 |  |  |  |
| *Vulnerable* | 1.19 | 0.69 | 2.04 | 0.533 |
| *Frail* | 1.59 | 0.98 | 2.59 | 0.062 |
| Age | 0.98 | 0.96 | 1.00 | 0.032 |
| Gender |  |  |  |  |
| *Male* | 1.00 |  |  |  |
| *Female* | 0.96 | 0.68 | 1.38 | 0.844 |
| Ethnicity |  |  |  |  |
| *White* | 1.00 |  |  |  |
| *South Asian* | 0.74 | 0.45 | 1.24 | 0.254 |
| *Black* | 0.75 | 0.44 | 1.27 | 0.291 |
| *Other* | 0.53 | 0.17 | 1.67 | 0.276 |
| BMI | 0.99 | 0.96 | 1.01 | 0.251 |
| IMD Quintile |  |  |  |  |
| *1* | 1.00 |  |  |  |
| *2* | 0.59 | 0.36 | 0.97 | 0.036 |
| *3* | 0.73 | 0.43 | 1.25 | 0.255 |
| *4* | 0.79 | 0.41 | 1.54 | 0.497 |
| *5* | 0.98 | 0.47 | 2.08 | 0.967 |
| *Unknown* | 1.15 | 0.58 | 2.31 | 0.686 |
| Charlson Index* | 1.11 | 0.99 | 1.25 | 0.079 |
| Previous admissions** | 1.12 | 1.03 | 1.22 | 0.007 |
| Number Medications | 1.00 | 0.95 | 1.06 | 0.997 |
| Smoking Status |  |  |  |  |
| *Current Smoker* | 1.00 |  |  |  |
| *Ex-Smoker* | 2.03 | 1.12 | 3.69 | 0.020 |
| *Never Smoked* | 1.30 | 0.76 | 2.21 | 0.341 |
| Albumin (g/L) | 0.96 | 0.92 | 0.99 | 0.013 |
| Walking Aid Use |  |  |  |  |
| *No* | 1.00 |  |  |  |
| *Yes* | 2.07 | 1.34 | 3.18 | 0.001 |
| HD vintage | 1.00 | 1.00 | 1.00 | 0.555 |
| Transplant Listing Status |  |  |  |  |
| *Not Active/Not Listed* | 1.00 |  |  |  |
| *Active* | 0.79 | 0.45 | 1.40 | 0.426 |
| Constant | 0.22 | 0.03 | 1.41 | 0.110 |

IRR= Incidence Rate Ratio, obtained by negative binomial regression. *=CKD omitted. **= Number of admissions in year prior to assessment.

**Supplementary Table S18: Fully adjusted negative binomial regression of total nights in hospital associated with CFS-MDT frailty status – ordinal variable**

|  | IRR | Lower 95% C.I. | Upper 95% C.I. | P |
| --- | --- | --- | --- | --- |
| CFS-MDT |  |  |  |  |
| *Robust* | 1.00 |  |  |  |
| *Vulnerable* | 1.65 | 1.01 | 2.69 | 0.045 |
| *Frail* | 1.91 | 1.19 | 3.07 | 0.008 |
| Age | 0.98 | 0.96 | 0.99 | 0.009 |
| Gender |  |  |  |  |
| *Male* | 1.00 |  |  |  |
| *Female* | 0.94 | 0.66 | 1.33 | 0.720 |
| Ethnicity |  |  |  |  |
| *White* | 1.00 |  |  |  |
| *South Asian* | 0.77 | 0.47 | 1.29 | 0.326 |
| *Black* | 0.81 | 0.48 | 1.36 | 0.424 |
| *Other* | 0.54 | 0.17 | 1.69 | 0.289 |
| BMI | 0.98 | 0.96 | 1.01 | 0.220 |
| IMD Quintile |  |  |  |  |
| *1* | 1.00 |  |  |  |
| *2* | 0.60 | 0.37 | 0.99 | 0.044 |
| *3* | 0.70 | 0.41 | 1.19 | 0.186 |
| *4* | 0.79 | 0.41 | 1.52 | 0.476 |
| *5* | 1.13 | 0.54 | 2.37 | 0.743 |
| *Unknown* | 1.08 | 0.54 | 2.17 | 0.833 |
| Charlson Index* | 1.10 | 0.98 | 1.23 | 0.122 |
| Previous admissions** | 1.13 | 1.04 | 1.23 | 0.005 |
| Number Medications | 1.01 | 0.95 | 1.06 | 0.781 |
| Smoking Status |  |  |  |  |
| *Current Smoker* | 1.00 |  |  |  |
| *Ex-Smoker* | 1.89 | 1.05 | 3.40 | 0.033 |
| *Never Smoked* | 1.28 | 0.75 | 2.17 | 0.370 |
| Albumin (g/L) | 0.97 | 0.94 | 1.01 | 0.117 |
| Walking Aid Use |  |  |  |  |
| *No* | 1.00 |  |  |  |
| *Yes* | 2.26 | 1.53 | 3.33 | <0.001 |
| HD vintage | 1.00 | 1.00 | 1.00 | 0.409 |
| Transplant Listing Status |  |  |  |  |
| *Not Active/Not Listed* | 1.00 |  |  |  |
| *Active* | 0.88 | 0.50 | 1.57 | 0.676 |
| Constant | 0.14 | 0.02 | 0.94 | 0.043 |

IRR= Incidence Rate Ratio, obtained by negative binomial regression. *=CKD omitted. **= Number of admissions in year prior to assessment.
